# Care Coordination for Mosunetuzumab Therapy in Patients With Follicular Lymphoma in Community Practices: Learnings From the MorningSun Study Investigators

**DOI:** 10.1002/cam4.70936

**Published:** 2025-05-28

**Authors:** Tara Graff, Ian Flinn, Jeff P. Sharman, Steven Liu, Bertrand M. Anz, Mitul Gandhi, Ayed Ayed, Richard Zuniga, Abdul Hai Mansoor, Lourenia M. Cassoli, Mei Wu, Prachi Jani, Juliana M. L. Biondo, Tony Lin, John M. Burke

**Affiliations:** ^1^ Mission Cancer and Blood Des Moines Iowa USA; ^2^ Tennessee Oncology/OneOncology Nashville Tennessee USA; ^3^ Willamette Valley Cancer Institute Eugene Oregon USA; ^4^ Alaska Oncology & Hematology LLC Anchorage Alabama USA; ^5^ Tennessee Oncology/OneOncology Chattanooga Tennessee USA; ^6^ Virginia Cancer Specialists Gainesville Virginia USA; ^7^ Cancer Specialists of North Florida Jacksonville Florida USA; ^8^ New York Cancer & Blood Specialists Port Jefferson New York USA; ^9^ Kaiser Permanente Portland Oregon USA; ^10^ Genentech, Inc. South San Francisco California USA; ^11^ Rocky Mountain Cancer Centers Aurora Colorado USA

**Keywords:** best practice, bispecific antibodies, care coordination, community practice, outpatient therapy

## Abstract

**Background:**

Preliminary data from the MorningSun study have demonstrated that outpatient subcutaneous mosunetuzumab can be safely administered.

**Aims:**

This publication describes how community centers in the MorningSun phase 2 study of outpatient subcutaneous mosunetuzumab in B‐cell non‐Hodgkin lymphomas prepared workflow and logistics (staff coordination, practice networks, and patient support) to monitor patients for cytokine release syndrome (CRS) and other toxicities.

**Materials and Methods:**

Ten investigators at US community practice study sites (one rural, seven urban, and two rural/urban) were interviewed between January 12 and February 22, 2024. Interview transcripts were analyzed qualitatively to identify key themes.

**Results:**

Prior to the study, 7/10 had limited/no experience administering bispecific antibodies for lymphoma. Regarding preparation before treatment, staff education was the most frequent need (7/10). All sites provided in‐service training for staff involved with treatment administration. Most respondents (6/10) had multidisciplinary plans and agreed these eased logistical concerns. Out of hours, patients either called the triage team, a dedicated on‐call number, the physician, or the emergency department. Most practices had preexisting relationships with hospitals for CRS management. All practices established methods for outpatient CRS monitoring; patient education and caregivers played important roles, and all respondents encouraged patients to use self‐monitoring devices. Each community practice had different workflow and logistics based on their setting and infrastructure.

**Conclusion:**

Community practices can leverage other sites' experiences and adopt an individualized approach to implementing bispecific antibodies safely and efficiently. Designating a physician champion could provide a local resource to address staff questions and concerns.

## 1 Introduction

Bispecific antibodies are a class of T‐cell engaging therapy that have shown promise in the treatment of B‐cell non‐Hodgkin lymphomas (B‐NHL) [1, 2]. Several bispecific antibodies have been approved for use in relapsed/refractory (R/R) follicular lymphoma (FL) or diffuse large B‐cell lymphoma (DLBCL) in the United States (US) [2, 3, 4, 5, 6]. These include fixed‐duration, outpatient, intravenously administered mosunetuzumab for R/R FL after ≥ 2 prior systemic therapies [2, 3, 6], fixed‐duration glofitamab [5] for R/R DLBCL after ≥ 2 prior systemic therapies, and epcoritamab for R/R DLBCL [4] or R/R FL after ≥ 2 prior systemic therapies, which is administered until disease progression [7].

Although bispecific antibodies have favorable overall safety profiles, treatment can be associated with toxicities, including cytokine release syndrome (CRS), infections, cytopenia, tumor flare, and neurologic adverse events (AEs) such as immune effector cell‐associated neurotoxicity syndrome (ICANS) [8]. CRS is a potentially life‐threatening toxicity that occurs due to increased immune activation and leads to elevated circulating cytokine levels [9]. CRS is the most frequently reported AE associated with bispecific antibodies for the treatment of R/R non‐Hodgkin lymphoma [8]. Although CRS events observed with bispecific antibodies are predominantly mild‐to‐moderate (grade 1/2) and manageable, more severe CRS has been reported (grade 3, 1.0%–3.0%; grade 4, 1.0%–1.1%) [3, 4, 5, 10]. To help mitigate risks, bispecific antibodies are administered with initial step‐up dosing to build up to target doses [4, 11, 12] and with premedications like corticosteroids, antihistamines, and/or antipyretics [4, 6, 13]. Tocilizumab is an anti‐cytokine medication that can be used for the treatment of CRS [9].

In a survey of healthcare professionals (HCPs; oncologists, nurses, pharmacists), 40% reported having no experience with the first approved bispecific T‐cell engaging therapy (blinatumomab) [1]. Most (86%) recognized the need for guidelines, shared best practices, and care recommendations to facilitate the adoption of blinatumomab. Transitioning from the inpatient to outpatient setting was identified as the key challenge. Consensus recommendations for managing bispecific antibody‐associated toxicities have recently been published [14]. The paper included logistical considerations such as: providing education to staff involved in administering bispecific antibodies; ensuring that a facility near a patient's address has access to tocilizumab to manage CRS; making clinical staff aware of tocilizumab location and method of administration; and advising patients to remain near this facility when at highest risk of CRS development.

MorningSun (NCT05207670) is an open‐label, multicenter, US‐based, phase 2 study of mosunetuzumab administered subcutaneously (SC) in patients with B‐NHL. In this trial, patients receive mosunetuzumab SC monotherapy without mandatory hospitalization in 21‐day cycles with initial step‐up dosing in Cycle 1. The mosunetuzumab SC injections used in this study contained higher mosunetuzumab concentrations than the intravenously administered preparation approved for use in R/R FL (10 or 45 mg/mL for SC injection versus 1 mg/mL for intravenous administration) [2, 3, 6]. The MorningSun trial completed enrollment in August 2024 and has an estimated study completion date of July 31, 2028. Preliminary efficacy and safety data have been previously reported and demonstrate that mosunetuzumab SC can be safely administered in an outpatient setting [15]. To participate in the MorningSun study, several community practices had to prepare to administer the bispecific antibody mosunetuzumab for the first time and manage potential CRS events; most physicians had little to no experience administering bispecific antibodies for lymphoma.

In order to understand how community practices prepared to deliver mosunetuzumab, we conducted a study in which we interviewed study investigators from community practices in the MorningSun study, gathering information about workflow, logistics, and toxicity management.

## 2 Methods

### 2.1 Study Design and Sampling

This qualitative interview study used semi‐structured, open‐ended interviews conducted with MorningSun study investigators to collect information about community practice logistics and workflow before administering mosunetuzumab in the outpatient setting. A total of 10 HCPs were selected for interviews between January 12 and February 22, 2024. Inclusion criteria were: community practice sites that enrolled ≥ 3 patients in the MorningSun study; no extensive experience with chimeric antigen receptor (CAR) T‐cell or bispecific antibody use in lymphoma in clinical studies or practice; and a newly established logistic/workflow for outpatient administration of mosunetuzumab. All inclusion criteria were required to be met for study participation. Sites were selected on September 29, 2023.

**FIGURE 1:**
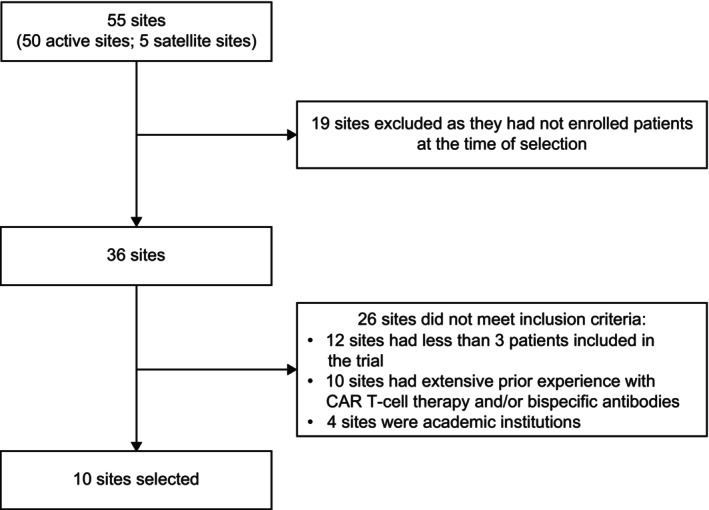
Flow diagram for site selection. CAR, chimeric antigen receptor.

This study used a purposive sampling strategy to select individuals who met all inclusion criteria and were able to provide relevant information; this involved selecting key contributors who generally had decision‐making authority and the most experience to contribute. Out of 55 study sites, 10 were selected, and principal investigators were interviewed to represent each site (Figure 1). Data was mainly collected during the interviews; however, during the development of this manuscript, additional insights were collected to support the information already obtained.

### 2.2 Data Collection

Two interviewers from the research department of a healthcare communications agency, Inizio Evoke, conducted individual 1‐h video interviews to capture HCP perspectives using an interview guide with prompts and follow‐up questions. There were no repeat interviews. The guide was pilot‐tested in the first interview and amended after the third interview to include additional follow‐up questions. The full interview guide can be found in the Supporting Information S1.

### 2.3 Data Analysis

Interviews were recorded and transcribed, and interviewers also made notes. Transcripts were not returned to participants for comments and/or correction, but participants reviewed findings during manuscript preparation. Interviews and transcripts were stored on Box and set to manually delete after 2 years.

Transcriptions were thematically analyzed using CoLoop, then manually analyzed in more detail by a senior research executive from a healthcare communications agency. Interview data were analyzed qualitatively to identify key themes. This paper presents an analysis of the major and minor interview themes.

### 2.4 Ethics Statement

Participants were informed that collected data would only be used for medical and scientific purposes. All participants provided informed consent before the start of the interview and had the opportunity to withdraw at any time. The protocol for the MorningSun study was approved by institutional review boards or ethics committees at each center. The study was conducted in accordance with the Declaration of Helsinki and applicable laws and regulations.

### 2.5 Data Sharing Statement

Data will be made available at reasonable request; please contact the corresponding author.

## 3 Results

### 3.1 Community Practice Characteristics

Ten US community practice sites located in Alaska, Colorado, Florida, Iowa, New York, Oregon, Tennessee, and Virginia, were included in this analysis (one rural community practice, seven urban community practices, and two rural/urban community practices; Table 1).

**TABLE 1:** Study site characteristics.

Institution	State	Setting	Size of institution			Experience with therapies prior to involvement in the MorningSun study	
			Number of physicians	Number of locations	Number of infusion chairs per site	Experience with bispecific antibodies	Experience with CAR T‐cell therapies
Alaska Oncology & Hematology LLC	Alaska	Rural	Five physicians, two physician assistants	1	17	No staff	No staff
Rocky Mountain Cancer Centers	Colorado	Urban	~55 oncologists	8	185 across all treatment sites	No staff	No staff
Cancer Specialists of North Florida	Florida	Urban	34 medical oncologists	12	11–26	No staff	No staff
Mission Cancer and Blood	Iowa	Urban	18 physicians, 27 APPs	Four urban‐based locations and 22 outreach clinics	100	No staff	No staff
New York Cancer & Blood Specialists	New York	Urban	75 oncologists	35	80	All staff	No staff
Kaiser Permanente	Oregon	Urban	Six hematology physicians	1	27	No staff	No staff
Willamette Valley Cancer Institute	Oregon	Urban	15 medical oncologists	6	60	Some staff	No staff
Tennessee Oncology	Tennessee (Nashville)	Urban and rural	85 medical oncologists across all locations	Two clinical administration sites among 20 research locations and 35 clinical locations	38	All staff	Some staff
Tennessee Oncology	Tennessee (Chattanooga)	Urban and rural	85 medical oncologists across all locations	Two clinical administration sites among 20 research locations and 35 clinical locations	30	All staff	No staff
Virginia Cancer Specialists	Virginia	Urban	40 physicians	10	12	No staff	No staff

Abbreviations: APP, advanced practice provider; CAR, chimeric antigen receptor.

The interviews with the 10 community practice sites included the following topics: workflow and logistics prior to bispecific antibody administration; staff and patient education; communication plans; and treatment administration, monitoring, and support.

### 3.2 Workflow and Logistics Prior to Bispecific Antibody Administration

All respondents were primary investigators (PIs) in the MorningSun study and were involved in implementing bispecific antibody treatment. Six respondents had no previous experience administering bispecific antibodies for lymphoma prior to the MorningSun study, one had some/limited experience, and three had prior experience.

In terms of workflow and logistics prior to outpatient treatment, staff education, including training on the management of CRS, ICANS, and other AEs, was the most frequently reported need (7/10). Other considerations noted were ensuring sufficient access to medications (e.g., tocilizumab and corticosteroids); establishing new and reviewing existing protocols; liaising with physicians involved in patient management; and establishing relationships with larger institutions. In addition, designating a physician champion to lead and coordinate the treatment program was suggested.

Seven respondents had preexisting relationships with institutions for patient admissions and CRS monitoring/management; designated hospitals were chosen due to existing collaborations or proximity. The hospitals were equipped with necessary staff and medications (like tocilizumab). Before administering treatment, staff at external hospitals were educated on managing potential AEs associated with bispecific antibodies. However, some respondents noted difficulties in educating external staff and establishing relationships. For the three respondents who did not have preexisting relationships, patient admissions, and CRS monitoring/management were maintained across various hospitals depending on proximity and/or availability. This approach relied heavily on ensuring that all institutions had access to necessary medication and staff to provide appropriate care.

In general, the importance of training was emphasized and was critical in mitigating concerns about patient care. Three respondents noted that nurses or oncologists without prior experience of bispecific antibodies may worry about outpatient treatment or worry that tocilizumab would reverse treatment effects. It was suggested that implementing a physician champion to address concerns would improve the transition to outpatient treatment with bispecific antibodies such as mosunetuzumab.

One small private community practice with five medical oncologists had no existing infrastructure before the study. The community site study team informed nearby hospitals about the trial and new treatment in advance and trusted that hospital staff would contact them if patients were admitted and they had questions about bispecific antibody therapy. At this practice, the team involved in the MorningSun study consisted of a medical oncologist and a physician assistant who oversaw the conduct of the study, including monitoring patients, providing education, and organizing logistics.

### 3.3 Staff and Patient Education

Physicians confirmed that all study sites provided in‐service training for staff involved with treating patients, in addition to the education provided by the MorningSun study team. Eight respondents gave staff further education, including an overview of mosunetuzumab and other bispecific antibodies, CRS monitoring and management, and other potential AEs. Half of respondents noted that education was tailored to staff members by their role, with some needing more in‐depth education than others.

Training was performed using a wide variety of tools and techniques. For staff, training included: in‐person initiation visits, online slide presentations, virtual training sessions, informal dinner meetings, phone calls, face‐to‐face sessions covering side effect monitoring and management, and information distribution through senior staff. For patients/caregivers, education focused on monitoring and managing side effects (such as CRS), when to take medications, and when and how to report symptoms; this was communicated through face‐to‐face training sessions, pamphlets, and wallet cards.

For most respondents, staff turnover was not an issue. However, for study sites that experienced staff turnover, education was maintained by leadership teams reassigning knowledgeable staff to fill gaps, and intensive staff training was provided along with allowing time to adapt to new roles.

### 3.4 Communication Plans

When asked what type of multidisciplinary communication plan was developed to support communication among site staff, six respondents confirmed they had a multidisciplinary plan in place, two of which predated involvement in the MorningSun study. Although four respondents did not implement a plan, most respondents felt that communication plans helped to ease the challenges of the logistical processes.

When creating a multidisciplinary communication plan, respondents considered both inpatient and outpatient treatment and drew on experience from previous clinical trials. Communication approaches varied across sites: six relied on digital communications (emails or phone calls), whereas one practice utilized regular multidisciplinary meetings. In general, communication plans were coordinated with emergency department (ED) and intensive care unit (ICU) staff.

Physicians found the most useful parts of a multidisciplinary communication plan were familiarity with protocols, study staff being available to other staff members and patients, and working as a team to maintain communication with patients and/or staff. Some challenges were raised, specifically ensuring staff engaged with plans, time required to educate HCPs on processes, and educating patients on sharing information with HCPs.

Across community practices, there was a varied approach to managing communication outside of working hours. Patients were either told to call a member of the research team directly (including the PI at one site), a dedicated triage number, an on‐call number, or the ED. One community practice site developed a patient buddy system. This patient‐led support program enabled consenting patients to contact each other, initially by email but then by phone or in person, depending on patient preferences. The first patient enrolled in this program was a little anxious about starting bispecific antibody treatment and later became an advocate, supporting other patients by sharing their experiences.

### 3.5 Treatment Administration, Monitoring, and Support

Across the community practices, a typical team administering bispecific antibodies included up to 10 nurses [including nurse managers; median: 5 (range: 1–10)], up to seven treating oncologists [median: 3 (range: 2–7)] providing direct care, other oncologists involved in overall care, a physician assistant (for some respondents), and a pharmacist (for some respondents). However, roles differed between practices when monitoring for side effects. Around half mentioned that clinical nurses, nurse managers, PIs, clinical research coordinators, and treating physicians were involved in CRS monitoring. Advanced practice providers, other oncologists, pharmacists, and physician assistants also contributed to CRS monitoring at three practices.

Physicians described parameters in place for monitoring CRS, noting that patients experiencing potential CRS symptoms could call the clinical team, the physician, PI, or the triage team. Nurses called and followed up with patients every 6‐h or daily during, and for 3 days after, step‐up dosing; twice a day if patients were admitted to the hospital during inpatient treatment; or “a few” times a week after initial dosing. Additional methods for enhancing CRS monitoring included educating patients on potential signs and symptoms of CRS, ensuring patients had monitoring equipment, and limiting the number of patients receiving treatment at the same time during step‐up dosing. Potential barriers to monitoring CRS and how they were overcome are outlined in Table 2.

**TABLE 2:** Addressing logistical challenges in CRS management.

Potential logistical challenges	Actions to overcome challenges
Staffing and resource coordination	
Forming a dedicated team of staff involved Coordinating availability of trained staff, due to rotations and shifts of staff involved Ensuring availability of necessary supplies, including critical medications like tocilizumab	Flexible patient enrollment plans were used, to allow for adjustments as comfort with treatment increased It was ensured that sufficiently trained staff were present at all sites Tocilizumab availability was proactively coordinated with hospitals ahead of time
Education and training challenges	
Patient adherence to CRS management protocols, especially regarding support and transportation Providing comprehensive team education for effective treatment logistics, including CRS management A lack of predefined communication plans among multidisciplinary teams	Targeted education strategies were implemented for both patients and staff to improve adherence Proactive planning allowed for thorough preparation and training was provided months in advance for all team members to be better prepared Specific educational needs of staff were expressed early on, and differences that were present due to infrastructure, location and/or capabilities were addressed
Workflow optimization and communication efficiency	
Lack of clear communication channels to allow swift responses during hospital admissions Developing an efficient IT system for patient admissions and management Lack of a clear workflow with key personnel where CRS was recognized quickly	Open lines of communication were established early in the process with all parties involved A reliable workflow was developed, where staff could recognize the signs and symptoms of CRS quickly

Abbreviations: CRS, cytokine release syndrome; IT, information technology.

The importance of having a caregiver was discussed. At nine sites, all patients had a caregiver. Patients with no caregiver were instructed to stay near the clinic for 3 days after step‐up dosing (which occurred only during Cycle 1). Caregivers attended appointments, received education, checked patient vital signs, and monitored for and reported side effects.

All respondents encouraged patients to use self‐monitoring devices (thermometers, blood pressure cuffs, oxygen saturation monitors, and heart rate monitors) and agreed that thermometers were the most important device. Some community practices provided devices to patients. One physician's community practice developed an app, which was monitored by the triage team in real time, where patients could self‐report side effects. Another respondent thought that logbooks or symptom trackers would be useful for patients.

Despite the range of devices and tools available, half of respondents noted that their site experienced challenges, including low patient adherence to recommendations (such as lack of engagement in monitoring or not reporting side effects), equipment costs, and patients attending clinic without caregivers. Generally, these concerns were overcome with education and discussions with patients (Table 3).

**TABLE 3:** Overcoming barriers for patients to self‐monitor symptoms and vital signs.

Barriers to self‐monitoring	Potential solutions
*Patient reluctance and preference* Patients dismissing the need to monitor their symptoms and vital signs Patient preference dictating whether they engage in self‐monitoring	*Education and training* Educating patients on recognizing signs and symptoms of CRS/AEs Communicating that self‐monitoring is crucial and non‐negotiable Emphasizing the importance of not managing symptoms independently at home
*Access to monitoring tools and technology* Cost and/or availability of monitoring tools Complexity of alternative solutions (such as apps) for patient engagement in self‐monitoring	*Access to resources* Providing patients with temperature monitors, oxygen saturation monitors, and other devices Alleviating costs for patients by manufacturers providing monitoring tools Streamlining data capture through technology or logbooks
*Physical capability and performance status* Eligibility for patient self‐monitoring	*Proactive patient selection and support strategies* Selecting patients who could reliably perform self‐monitoring tasks Monitoring and providing support to high‐risk patients
*Support system limitations* Lack of caregiver involvement impacting patient self‐monitoring Distance from the hospital delaying access to medical care if needed Transportation issues preventing timely access to medical assistance	*Team coordination and communication* Coordinating nurse triage systems and case management Communicating multidisciplinary team plans Educating healthcare teams on the importance of self‐monitoring Advising patients to stay near a hospital while receiving treatment

Abbreviations: AE, adverse event; CRS, cytokine release syndrome.

Physicians were asked if they requested that patients stay near clinics after treatment. Eight respondents required patients to stay near the clinic during step‐up dosing (five required patients to stay within 45 min or less, and three required patients to stay within 1‐h). Despite this, patients occasionally visited other institutions, leading to concerns such as unknown access to tocilizumab, limited control of patient care, and hospital staff who may not have experience with bispecific antibody therapies. One site set up an IT system flag alert notifying treating hospitals when patients receiving bispecific antibody therapy were admitted to non‐designated hospitals within the provider's healthcare system. Research nurses could then follow up and request details of the hospitalization/ER visit.

### 3.6 Key Considerations for Community Practices

**FIGURE 2:**
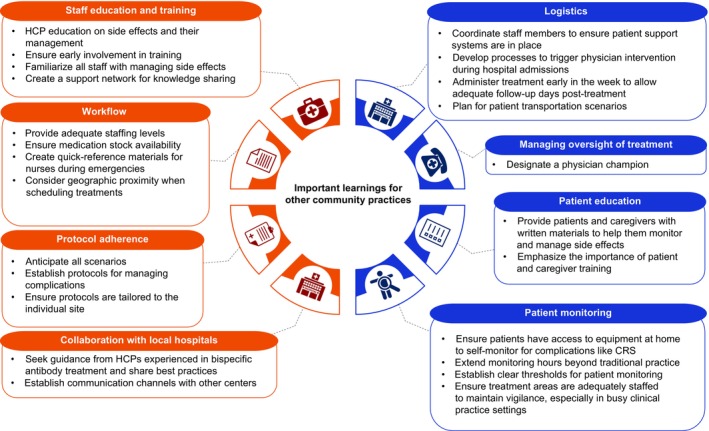
Key insights for community practices wanting to begin outpatient treatment with bispecific antibodies such as mosunetuzumab. Orange represents staff training and coordination prior to mosunetuzumab dosing. Blue represents safe outpatient monitoring after mosunetuzumab dosing. CRS, cytokine release syndrome; HCP, healthcare professional.

During the interviews, physicians outlined practical considerations for administering bispecific antibodies in community practices without extensive prior experience with these agents. Figure 2 shows a detailed list of the insights from the interview. The most popular considerations that emerged were: (1) designating a physician champion to respond to any patient and/or staff concerns and oversee education was deemed critical; (2) gaining experience using bispecific antibodies improved the physicians perception of treatment use and lessened concerns around side effects and tocilizumab use; (3) ensuring that clinics and hospitals maintained sufficient stocks of tocilizumab was essential, especially in more rural settings; (4) familiarizing staff with relevant protocols and having clear communication channels helped treatment coordination run smoothly; and (5) prioritizing staff, patient, and caregiver training was essential for monitoring for side effects. Another notable consideration was administering treatment early in the week to avoid side effects occurring during out‐of‐office hours and to allow adequate follow‐up days post treatment.

## 4 Discussion

In this qualitative study, physicians enrolling patients for outpatient mosunetuzumab SC treatment gave perspectives on how their community site organized logistics and workflow before and during treatment, and also considerations for community practices who may administer mosunetuzumab SC in the future. Overall, first‐hand experience with bispecific antibodies reduced concerns around their use and side effects, as well as around tocilizumab treatment for CRS. The respondents' experience and recommendations were only reflective of the use of mosunetuzumab SC.

In line with the literature [1, 14], there was consensus that education was critical before administering bispecific antibodies, specifically training on management of CRS, ICANS, and other AEs. Successful education can make patients more comfortable and staff more confident with treatment, which is important when transitioning from inpatient to outpatient administration [1]. Training on the management of side effects associated with T‐cell engaging therapies can extend outside the treatment team to ED and ICU staff [16]. However, in this study, some respondents encountered difficulties in educating staff of external hospitals and establishing new relationships. Designating a physician champion could overcome these issues by providing support where needed. For community practices without experience with bispecific antibodies, the authors recommend focusing on training staff, including external staff in partnered hospitals, and designating a physician champion to provide oversight.

Expert consensus recommendations suggest that patients and caregivers are provided with educational resources on monitoring and management of toxicities associated with bispecific antibodies, including information about AEs, healthcare team contact information, instructions for self‐monitoring, and wallet cards [14]. Physicians in the current study reported the use of similar tools and techniques. These resources may be useful to standardize and implement across community practices where bispecific antibodies are administered. Similarly, in outpatient programs for other treatments, leaflets/information cards and checklists for infusion nurses [17] or patient wallet cards [17, 18] were often provided to supplement education. Community practices looking to implement bispecific antibody treatment could focus on educating patients on the signs and symptoms of CRS and other toxicities, how to self‐monitor, and when/who to contact if symptoms of toxicity arise.

Relationships with designated hospitals have been highlighted as important to allow swift communication and inpatient support where needed [14]. The expert consensus recommendations for outpatient bispecific antibodies [14] and previous studies of CAR T‐cell therapies [16] emphasize the importance of having relationships with hospitals and other HCPs, whose expertise can be leveraged to treat patients with toxicities. Three sites in the current study did not have a preexisting relationship with a designated hospital before initiating treatment, demonstrating that administration of bispecific antibodies is possible, regardless of location and/or current infrastructure.

Most physicians agreed that having a multidisciplinary plan in place for this study eased logistical concerns, but the approach taken varied across community sites. This is consistent with outpatient CAR T‐cell therapy studies, which suggested establishing a plan for clinical, ED, and other staff [18]. Community practices can leverage communication plans to familiarize staff with protocols and provide reassurance; a physician champion could ensure that the multidisciplinary plan is in place and distributed where needed.

Since patients sometimes visited non‐designated institutions, concerns were raised regarding access to tocilizumab, limited control over patient care, and the possibility that staff were not educated about bispecific antibody treatment. To address this, some community sites provided patients with identification cards so external staff could contact the research team; one site set up an alert if a patient was admitted to an external hospital. This approach is supported by a framework established for outpatient CAR T‐cell therapy, where patients' electronic medical records alerted ED staff that the patient is on CAR T‐cell therapy and whom to contact [16]. In terms of future considerations, patients can be guided to designated hospitals where possible, although protocols could be put in place for alternative scenarios.

In conclusion, although each community practice had different protocols, all sites agreed that by setting up a team of dedicated HCPs, prioritizing staff and patient/caregiver education, and creating a support network of clinics/hospitals, patients could receive outpatient mosunetuzumab therapy while being thoroughly monitored for CRS and other AEs. Community practices wanting to initiate bispecific antibody therapy can leverage the experience of other community sites when creating their own protocols and adopt a tailored approach to integrating bispecific T‐cell engaging therapies safely and efficiently into their clinical practice. Designating a physician champion is crucial for oversight of bispecific antibody programs and to act as a local resource to address staff questions and concerns. With appropriate care coordination and protocols in place, community practices can successfully implement and administer mosunetuzumab SC to their patients in an outpatient setting.

## Author Contributions

**Tara Graff:** conceptualization (equal), formal analysis (equal), methodology (equal), resources (equal), writing – original draft (lead), writing – review and editing (equal). **Ian Flinn:** conceptualization (equal), formal analysis (equal), methodology (equal), resources (equal), writing – original draft (equal), writing – review and editing (equal). **Jeff P. Sharman:** conceptualization (equal), data curation (equal), formal analysis (equal), methodology (equal), resources (equal), writing – original draft (equal), writing – review and editing (equal). **Steven Liu:** data curation (equal), formal analysis (equal), resources (equal), writing – original draft (equal), writing – review and editing (equal). **Bertrand M. Anz:** data curation (equal), formal analysis (equal), resources (equal), writing – original draft (equal), writing – review and editing (equal). **Mitul Gandhi:** data curation (equal), formal analysis (equal), resources (equal), writing – original draft (equal), writing – review and editing (equal). **Ayed Ayed:** formal analysis (equal), resources (equal), writing – original draft (equal), writing – review and editing (equal). **Richard Zuniga:** resources (equal). **Abdul Hai Mansoor:** conceptualization (equal), formal analysis (equal), methodology (equal), resources (equal), writing – original draft (equal), writing – review and editing (equal). **Lourenia M. Cassoli:** formal analysis (equal), writing – original draft (equal), writing – review and editing (equal). **Mei Wu:** conceptualization (equal), formal analysis (equal), methodology (equal), writing – original draft (equal), writing – review and editing (equal). **Prachi Jani:** conceptualization (equal), data curation (equal), formal analysis (equal), methodology (equal), resources (equal), writing – original draft (equal), writing – review and editing (equal). **Juliana M. L. Biondo:** conceptualization (equal), data curation (equal), formal analysis (equal), methodology (equal), writing – original draft (equal), writing – review and editing (equal). **Tony Lin:** conceptualization (equal), data curation (equal), formal analysis (equal), methodology (equal), writing – original draft (equal), writing – review and editing (equal). **John M. Burke:** formal analysis (equal), resources (equal), writing – original draft (equal), writing – review and editing (equal).

## Ethics Statement

The protocol for the MorningSun study was approved by institutional review boards or ethics committees at each center. The study was conducted in accordance with the Declaration of Helsinki and applicable laws and regulations.

## Consent

Participants were informed that collected data would only be used for medical and scientific purposes. All participants provided informed consent before the start of the interview and had the opportunity to withdraw at any time.

## Conflicts of Interest

T.G. has served as a consultant or advisor for AbbVie, Adaptive Biotechnologies, AstraZeneca, Genentech, Inc., Genmab, and TG Therapeutics; a member of advisory boards or steering committees for AbbVie, Bristol Myers Squibb, BeiGene, Genmab, Gilead Sciences, and Eli Lilly; and a speaker for AbbVie, Adaptive Biotechnologies, ADC Therapeutics, AstraZeneca, BeiGene, Genentech, Inc., Genmab, Gilead Sciences, Lilly, and TG Therapeutics. I.F. reports consultancy with AbbVie, BeiGene, Genentech, Inc., Genmab, Kite Pharma, and Vincerx Pharma; has received research funding from 2seventy bio, AbbVie, AstraZeneca, BeiGene, Bristol Myers Squibb, Celgene, City of Hope National Medical Center, Epizyme, F. Hoffmann‐La Roche Ltd., Fate Therapeutics, Genentech, Inc., Gilead Sciences, IGM Biosciences, Incyte, InnoCare Pharma, Janssen, Kite Pharma, Loxo, Marker Therapeutics, Merck, MorphoSys, Myeloid Therapeutics, Novartis, Nurix, Pfizer, Seattle Genetics, TG Therapeutics, and Vincerx Pharma; and has served as a member of a Vincerx Pharma Advisory Committee. J.P.S. reports consultancy for AbbVie, AstraZeneca, BeiGene, Bristol Myers Squibb, Lilly, Merck, Genmab, and Genentech, Inc. S.L. holds equity in Bristol Myers Squibb, Lilly, and Pfizer; and has received research funding from Bristol Myers Squibb, F. Hoffmann‐La Roche Ltd., Genmab, Gilead Sciences, Lilly, Novartis, and Pfizer. B.M.A. reports current employment with Tennessee Oncology; all research is conducted through, and funding is paid directly to, the Sarah Cannon Research Institute. M.G. has received honoraria from GSK, Janssen, Karyopharm Therapeutics, and TG Therapeutics; and has served as a member on advisory boards for GSK, Janssen, Karyopharm, Sanofi, and TG Therapeutics. A.A. reports consultancy for Bristol Myers Squibb, Genentech, Inc., Ipsen, and Janssen. R.Z. has served as a consultant or advisor to Bristol Myers Squibb and Mirati Therapeutics. A.H.M. declares no competing financial interests. L.M.C. is an employee of and holds equity in Genentech, Inc. M.W. is an employee of Genentech, Inc., and holds equity in F. Hoffmann‐La Roche Ltd. P.J. is an employee of Genentech, Inc., and holds restricted stock units in F. Hoffmann‐La Roche Ltd. J.M.L.B. is an employee of Genentech, Inc., and holds equity in and has received honoraria from F. Hoffmann‐La Roche Ltd. T.L. has received grants paid to his institution from BeiGene, and has participated on data safety monitoring boards or advisory boards for AstraZeneca, Boehringer Ingelheim, MSD, Servier, and Transcenta. J.M.B. reports consultancy with AbbVie, AstraZeneca, BeiGene, Bristol Myers Squibb, F. Hoffmann‐La Roche Ltd., Foresight Diagnostics, Genentech, Inc., Genmab, Novartis, Regeneron, and Seagen.

## Supporting information


Data S1.

## Data Availability

Data will be made available at reasonable request; please contact the corresponding author.
