# Supplementary material for: Care Coordination for Mosunetuzumab Therapy in Patients With Follicular Lymphoma in Community Practices: Learnings From the MorningSun Study Investigators
Source: Cancer Med. 2025 May 28;14(11):e70936. doi: 10.1002/cam4.70936 (PMC12117195; doi:10.1002/cam4.70936)
Supplement: Supplementary file 1 — Data S1. [file CAM4-14-e70936-s001.pdf]

evoke mind+matter

MorningSun Interviews

60-minute web-assisted telephone interviews

Discussion Guide

Version 4.0

Contact:

Josie Stratton
josie.stratton@evokegroup.com

Rachel Butcher
rachel.butcher@evokegroup.com

MorningSun Interviews

**Title: MorningSun Interviews**<br>Author: Josie Stratton<br>Version: v3.0
**Objectives:**<br>• Understanding community practice preparations for outpatient administration throughout the MorningSun Phase II study
**Method:** 60-minute telephone depth interview (TDI)
**Quotas:** N=11 personnel from the MorningSun Phase II study

Pronunciation - Mosunetuzumab [*Moe-sun-et-oo-zuh-mab*]

Section 1 – Introduction (3 minutes)

Good morning/afternoon/evening. My name is ______________________ and I am working on behalf of Inizio Evoke Europe who are the agency commissioned to conduct this research on behalf of Genentech.

OBJECTIVE:
This discussion will take 60 minutes and will focus on how your community practice prepared in terms of logistics and workflow, before joining the MorningSun Phase II study. The following areas of care coordination are of particular interest: staff coordination, practice network, and patient support.

The findings of these discussions will support development of a manuscript that will share different logistics and workflow setup experiences of community sites before the use of a bispecific antibody treatment (mosunetuzumab subcutaneous/SC) in the clinical trial setting. However, the totality of the experiences in the manuscript will inform other community sites across the country that there is no one-size-fits-all approach when preparing their practice for the use of bispecific.

AUDIO RECORDING:
We are being **audio recorded** to make sure I capture all of your responses and to be able to refer to them during analysis. The recordings will be stored for up to 2 years and will only be available to the teams at Inizio Evoke Europe and Ashfield MedComms. Before we start, please can you re-confirm that you are willing for our discussion to be recorded?
Get consent and close if respondent says no

MorningSun Interviews

CLIENT SHARING:
Members at Genentech have expressed interest in listening to some of these interview recordings. The recordings would only be shared with relevant contacts at Genentech, within medical affairs team roles, and they will only use the information shared for medical and scientific purposes. Please can you confirm whether you are willing for your recording to be shared with Genentech?

ADVERSE EVENT REPORTING:
During the course of our discussion, you may happen to mention that participants from the MorningSun Phase II study experienced side effects, product complaints or special reporting situations when receiving the mosunetuzumab SC treatment. If this occurs, we are required to pass this information onto Genentech, even if the side effect is something that has previously been reported. Are you happy to proceed on this basis?
Get consent and close if respondent says no

Section 2 – Background (2 minutes)

To ease respondents into the interview and understand their professional background.

1. To get us started, please can you tell me a bit about your professional background, including your current role and what setting you work in?
    a. How long have you been in your current role?
    b. How is your practice structured? For example, are you partnered with a hospital or another organisation, etc.
    c. What type of patients do you see? Please think about therapy areas or demographic details.
    d. Important – What was your role in implementing new treatments at your institution before joining the MorningSun study?
        i. Were you the key decision maker? If not, who was?
        ii. [If the PI was not the one leading the implementation] Who was the point person leading the implementation of treatments?

Section 3 – Staff Coordination (19 minutes)

Exploring staff involvement and responsibilities during the MorningSun study.

This first section of questions will be about staff coordination, training, and involvement in the MorningSun trial.

2. Considering workflow and logistics, what was needed prior to joining the study to ensure that you had all the capabilities needed to take part in the trial?
    a. Were any adjustments made to standard clinical practice to support your trial involvement?

evoke mind+matter

MorningSun Interviews

3. How are patients at your clinic monitored when receiving a new bispecific antibody treatment such as mosunetuzumab? [Probe around administering mosun earlier in the week so CRS is less likely over the weekend, having a nurse call the patient the next day to check the patient's temperature]
    a. What call parameters are in place for CRS monitoring?
        i. How often does the nurse call on patients with CRS?
    b. Which staff members are typically involved in CRS monitoring? What are their key responsibilities?
    c. Is there a specific team set up to handle bispecific treatments?
        i. Which staff members make up this team?
        ii. Does the core team remain the same for all patients receiving bispecifics?

4. Beyond education related to the study protocol (for example, mosunetuzumab administration, monitoring adverse events, CRS management, etc), did you provide staff with any education on bispecific antibody drugs, either at a class level or on a specific adverse event such as CRS?
    a. Did the education differ based on the role of the staff member? In what way?
    b. Did you develop an additional document/algorithm to help support your site staff?
        i. What information was included in this document?
    c. Important - If you faced high staff member turnover, how did you maintain education levels?
    d. Was in-service training provided before the first patient was enrolled?

5. What type of multidisciplinary team communication plan was developed to support communication among site staff and with other departments if/when needed?
    a. What were the three most useful parts of this plan?
    b. What did this plan look like for after-hours care?
    c. Was there a specific plan to triage patients' calls?
    d. Please describe any challenges that arose while implementing this plan.
        i. How were these challenges resolved?
        ii. Is there anything that you would do differently when resolving these challenges?
    e. When developing this plan, did you need to consider inpatient and outpatient team coordination?
        i. What does this coordination look like?
    f. [If no plan was developed] Why was a communication plan not developed?

6. In your experience, who were trial patients at your clinic educated to contact if they were experiencing any adverse events?
    a. Did this change depending on the time of day?

        i. [If yes] Who would they contact outside of office hours?
    b. Were caregivers provided with any different or additional training to patients?

7. Is there a specific process or part of the process you implemented to improve monitoring and management of CRS that you feel will be useful for future patients?

8. What practical considerations should be made for community physicians with no experience of bispecific antibody treatments who will be using mosunetuzumab for the first time?
    a. Which of these is the most important?

Section 4 – Practice Network (12 minutes)

Understanding existing networks with other clinics, as well as any developed specifically for the MorningSun trial.

We're now going to talk a bit about any connections or collaborations you had with other facilities, such as hospitals, emergency departments, and network or satellite clinics, both before and during the MorningSun trial.

9. Did you have a designated hospital for patient admissions for CRS monitoring or management prior to joining the trial?
    a. [If yes] When forming this collaboration, who did you talk to in the partnering hospital to formalise this process? For example, members of hospital management, clinical staff, etc.
    b. [If yes] Did you face any challenges when establishing or maintaining this collaboration?
    c. [If yes] Have you ever needed to provide any kind of education for the hospital staff?
        i. Was in-service training provided?
        ii. How were hospital staff trained about the MorningSun trial protocol and CRS management?
    d. [if no] What criteria did you use to select hospitals for patient referrals during the trial, in case hospitalization was needed?
        i. Did you experience any challenges when collaborating with other hospitals?
            - [If yes] How were these resolved?

10. Which members of staff from collaborating hospitals/clinics did you talk with, in order to formalise your partnership? [Probe around hospital management, clinical staff]

11. Will you continue these collaborations once the trial has concluded?
    a. Why/why not?

Section 5 – Patient Support (18 minutes)

Discussing pre- and post-treatment support received by patients and caregivers.

I'd now like to ask you some questions about CRS monitoring and management guidance given to patients and caregivers during the MorningSun trial.

12. What training or education was provided to patients or caregivers ahead of starting mosunetuzumab SC treatment?
    a. [If yes] Did you provide any training beyond that provided by the study team?
        i. [If yes] What did this training include? Why did you feel it was necessary?

13. Were patients encouraged to interact with one another during the trial?
    a. [If yes] Did patients support one another through the trial? How?

14. What was the role of patient caregivers during the mosunetuzumab treatment journey?
    a. Did caregivers tend to be more receptive to or engaged with education than patients?
    b. Did you have any patients who did not have a caregiver?
        i. Did patients without caregivers receive different CRS monitoring training?
        ii. [If yes] How did patients without caregivers monitor signs and symptoms at home?
        iii. [If yes] How did patients without caregivers communicate with site staff to request assistance if needed?

15. Were patients required to stay near the clinic during step-up dosing?
    a. Did requirements differ for patients who did not have a caregiver?

16. In your experience, which tools did patients use to track symptoms and vital signs at home?
    a. Did you provide any CRS monitoring materials to patients or caregivers beyond those provided by the study team?
    b. In your opinion, which tool was most useful?
    c. Will you continue to use/recommend any of these tools after the trial ends?
        i. Which tool(s) will you continue to use with future patients?

17. Were there any barriers preventing patients from self-monitoring symptoms and vital signs?
    a. [If yes] What barriers prevented this?
    b. [If yes] How were these overcome?

Section 6 – Final thoughts (5 minutes)

Gaining a final perspective on what was gained from the MorningSun study.

I'm now going to ask you some final questions on what you took away from the MorningSun study and any difficulties you had to overcome. When answering these questions, we'd like you to think about the logistical aspects of your practice, before and during the study rather than clinical data.

18. What was the biggest hurdle that you faced in relation to CRS logistics setup and management?
    a. How did you overcome this?

19. What are the most important learnings to share with pharmaceutical companies or community practices about setting up new CRS-related logistics and workflow processes for the use of a bispecific antibody?
    a. In your opinion, will these learnings continue to inform processes at your clinic in the future?
        i. How?

Section 7 – Close (1 minute)

Before I end the session, is there anything else that you would like to add about what we have spoken about today?
